# Maternal and Child Health, Non-Communicable Diseases and Metabolites

**DOI:** 10.3390/metabo13060756

**Published:** 2023-06-15

**Authors:** Marlon E. Cerf

**Affiliations:** 1 Grants, Innovation and Product Development, South African Medical Research Council, P.O. Box 19070, Tygerberg, Cape Town 7505, South Africa; marlon.cerf@mrc.ac.za; 2 Biomedical Research and Innovation Platform, South African Medical Research Council, P.O. Box 19070, Tygerberg, Cape Town 7505, South Africa

**Keywords:** cancer, cardiovascular disease, developmental programming, diabetes, gut microbiota, mental illness, obesity

## Abstract

Mothers influence the health and disease trajectories of their children, particularly during the critical developmental windows of fetal and neonatal life reflecting the gestational–fetal and lactational–neonatal phases. As children grow and develop, they are exposed to various stimuli and insults, such as metabolites, that shape their physiology and metabolism to impact their health. Non-communicable diseases, such as diabetes, cardiovascular disease, cancer and mental illness, have high global prevalence and are increasing in incidence. Non-communicable diseases often overlap with maternal and child health. The maternal milieu shapes progeny outcomes, and some diseases, such as gestational diabetes and preeclampsia, have gestational origins. Metabolite aberrations occur from diets and physiological changes. Differential metabolite profiles can predict the onset of non-communicable diseases and therefore inform prevention and/or better treatment. In mothers and children, understanding the metabolite influence on health and disease can provide insights for maintaining maternal physiology and sustaining optimal progeny health over the life course. The role and interplay of metabolites on physiological systems and signaling pathways in shaping health and disease present opportunities for biomarker discovery and identifying novel therapeutic agents, particularly in the context of maternal and child health, and non-communicable diseases.

## 1. Introduction

The gestational–fetal period is a critical stage when maternal and fetal health is vulnerable (i.e., during pregnancy (mother) and fetal development (child)), which extends to the lactational–neonatal period (i.e., during lactation and early life, respectively), and these dual life phases, for mother and child, require a healthy milieu to (i) maintain maternal health and well-being and (ii) for the conveyance of beneficial maternal sequelae for optimal fetal and neonatal growth, development, physiology and metabolism. Developmental programming refers to stimuli or insults derived in utero and/or in early neonatal life, influenced by mothers, which shape fetal and neonatal outcomes, and often have immediate and persistent effects on progeny over their life course [1]. Therefore, a healthy mother—preconception and during gestation and lactation—offers her child the best possible growth, development and health outcomes, which are determined by genetic and environmental cues.

Non-communicable diseases yield high morbidity and mortality, and they hold high prevalence and incidence globally, particularly the cluster of diabetes, cardiovascular disease and cancer, which diminish quality of life through evolving etiologies and lengthy and costly durations of treatment. Metabolomics can be utilized to characterize metabolic derangements and phenotypes that lead to non-communicable diseases [2], and to identify biomarkers and novel targets for diagnosis and the monitoring of therapeutic activity [3]. Further, metabolomics reveals insights into environmental factors (e.g., nutrition) [4], and many metabolites are derived from nutritional metabolism. Metabolites can be utilized for biomarker discovery to identify risks for diseases early, as they reflect a snapshot of diverse metabolic functions [5]. Altered metabolite profiles arise from nutrition, and with infrequent physiological events that can unravel potential mechanisms and novel approaches to prevent and treat diseases [6].

Metabolites reflect various metabolic pathways, their interactions, and genetic and environmental exposures [7]. Further, metabolites shape health and disease; for example, specific micronutrients are required to maintain maternal health and for healthy fetal development, as a lack of specific amino acids, the over-consumption of certain undesirable fatty acids or excessive glucose can lead to the onset of non-communicable diseases. Amino acids, fatty acids (and glycerol) and monosaccharides (i.e., glucose, galactose and fructose) are monomers of proteins, lipids and carbohydrates, respectively. After ingestion and digestion, food is broken down into monomers (and nutrients) (i.e., metabolized) to provide metabolites (and energy) that are integral for metabolic signaling for maintaining physiology and metabolism (in mothers and children). Nutritionally derived metabolite sources drive progeny growth and developmental trajectories as the stimuli or insults that shape the progeny’s health outcomes, which links to the developmental programming of health and disease. Thus, metabolites shape health and disease, and disentangling their interplay and roles holds predictive potential for maintaining health by preventing the onset of disease. This review presents, in the context of maternal and child health, (i) an overview of non-communicable diseases, mainly diabetes and cardiovascular disease, with some insights on cancer, mental illness and obesity; and (ii) highlights the role and importance of metabolites in non-communicable diseases.

## 2. Maternal and Child Health and Developmental Programming

Fetal growth, development, physiology and metabolism are dependent on nutrient transport through the placenta; thus, maternal metabolism adapts to compensate substrate over-utilization [8] during the gestational–fetal phase. An adequate essential fatty acid supply in gestation and early neonatal life is key for healthy fetal growth, development, physiology and metabolism. Polyunsaturated fatty acids, derived from maternal nutrition, may alter epigenetic gene regulation by the transcriptional induction or repression of key genes during ontogeny [9,10] that influence progeny health over the life course. Lipids are multifunctional and ubiquitous metabolites and are often essential for various cell and organism functions [11]. Fatty acids serve as substrates to produce energy, and specific fatty acids trigger various signaling pathways for cellular function [12]. In fetal rats (whose mothers were fed high-fat diets), the plasma linoleic and arachidonic acid profiles were increased [13], and their mothers had elevated hepatic stearic and oleic acid profiles, increased adipose tissue stearic acid and oleic acid profiles and increased placental neutral myristic acid profiles [14], reflecting the variances in high-fat diet metabolism in fetal and maternal plasma and organs. Exposure to elevated glucose concentrations (i.e., hyperglycemia) in utero, for example, could lead to hyperglycemia in neonatal progeny [15] over the life course. In addition, elevated saturated fatty acids can lead to diabetic phenotypes, whereas protein deficiencies (altered amino acid profiles) in utero can lead to intrauterine growth restriction and predispose to diabetes and cardiovascular disease later in life. This reflects the importance of metabolites (e.g., monomers) for maintaining physiology and preventing diseases in mothers and children.

Glucose flows from the mother to the fetus as the main energy source for growth and development, and it is generated mainly from carbohydrate dietary sources and gluconeogenic amino acids [16,17,18,19]. Elevated maternal glycemia was linked to higher birth weights and lower duration of pregnancy (and increased risk of preterm delivery) [19]. There is an increased risk for fetal growth restriction when, in late gestation, the 2 h oral glucose tolerance test (OGTT) is ≤fasting glycemia [20]. Glucose-intolerant pregnant women usually carry heavier fetuses (macrosomia) [20], which may predispose their progeny to childhood obesity that persists into adulthood. Thus, glucose concentrations, during the gestational–fetal phase, are a key determinant of maternal and child health outcomes, due to the influence on pregnancy duration, delivery and birth weights, and, through fetal programming, hyperglycemia can predispose progeny to diabetes and cardiovascular disease later in life.

## 3. Diabetes, Cardiovascular Disease and Metabolites

In gestation and lactation, mothers need increased protein intakes for tissue deposition and milk production [21]. Isoleucine, leucine, valine, glutamine and tyrosine were linked to both diabetes prevalence and incidence, whereas lysine, phenylalanine, alanine, glutamic acid and glycine were associated with diabetes prevalence, and tryptophan was associated with diabetes incidence [22]. Valine and tyrosine independently improved in diabetes incidence risk prediction [22]. In people with diabetes, leucine, lysine, phenylalanine, tryptophan, aspartate, glutamate and cystine were increased, whereas alanine and serine were reduced and correlated with fasting blood glucose and HbA1c, but not with lipid profiles [23]. In streptozotocin-induced type 1 diabetic mice, supplementation with the amino acid derivative taurine ameliorated hyperglycemia and insulin resistance [24]. Therefore, amino acids play a role in shaping the diabetogenic phenotype by influencing glycemia.

In diabetes, unravelling the key factors that regulate glycemia in prediabetes towards achieving normoglycemia can lead to the prevention of the onset of hyperglycemia and, consequently, diabetes [4]. Specific metabolites shaped the glucose regulation in prediabetic people following 24 weeks (~5.5 months) of lifestyle intervention (hypocaloric diet and >150 min of physical activity per week) [4]. Dynamic altered metabolite levels in an OGTT were linked to cardiovascular disease and mortality and were relevant for the earlier identification of the cardiovascular disease risk and the discovery of novel therapeutic targets [5]. Metabolic responses to OGTTs, therefore, could identify high-risk individuals prior to the emergence of overt risk factors and can present targets for the earlier prevention of cardiovascular and cardiometabolic disease [5]. Defining the metabolite fluctuations during OGTTs could yield insights into the metabolite interplay during a glucose challenge, reaching glucose homeostasis, and reveal metabolites implicated in the onset of impaired glucose tolerance, insulin resistance and beta cell dysfunction that precede the onset of overt diabetes.

Polyamine metabolism dysregulation influences lipid, glucose and energy homeostasis, with high circulating putrescine (a polyamine found in fruit, vegetables and seeds) in diabetes patients, as putrescine was positively correlated with HbA1c, reflecting the adverse role of putrescine in glucose regulation, and thus low putrescine levels were linked to an improvement towards normoglycemia [4]. The hypoxanthine 7-methylguanine was linked to the regression to normoglycemia [4]. Participants with increased protein consumption were more likely to have normal glucose tolerance relative to those who maintained their protein consumption [4]. A high-protein diet was reported to result in the 100% remission of prediabetes, compared to 33% for high-carbohydrate dietary consumption [25]. Increases in gastric inhibitory polypeptide, glucagon-like peptide-1 and ghrelin (incretin hormones (i.e., gut hormones that are secreted after ingestion)) in a high-protein diet improve insulin sensitivity and beta cell function [26]. After a lifestyle intervention, there was an increased plasma abundance of taurine and hypotaurine, which are anti-inflammatory and antioxidant metabolites [27], and there was reduced glucose concentrations to reflect improvements in hyperglycemia and insulin resistance [28]. Alterations in N-acetyl-D-galactosamine, putrescine and 7-methylguanine were linked to regression to normoglycemia, which was independent of the baseline HbA1c and weight loss [4]. Further, higher protein consumption could favorably impact the treatment of prediabetic people [4]. High-protein diets may be beneficial in prediabetes regression to normoglycemia and greater insulin sensitivity, but further studies are required in different populations with diverse metabolic phenotypes, and the consideration of other macronutrients, micronutrients and metabolites will provide further insights into diabetes prevention. Novel metabolites could be investigated in the context of developmental programming to ascertain child growth, development, physiology and metabolism and the roles and interplay of metabolites. Biomarkers derived from metabolite profiles may inform the early onset or pathogenesis of some non-communicable diseases.

Gut microbiota is a source for metabolites and is associated with diabetes and cardiovascular disease. The gut microbiota composition plays a role in the pathogenesis of some non-communicable diseases (e.g., diabetes, cardiovascular disease and obesity), but there is limited research on the causality and mechanisms of action [29,30,31]. Gut microbiota accounted for ~58% of the variances in individual plasma metabolites; there were 997 links of metabolites and alpha diversity, with 546,819 associations between metabolites and specific gut microbiota [32]. Gut microbiota is influenced by a person’s lifetime exposure to diet, diseases, treatments (e.g., antibiotics) [33], genetic variation and age [34]. A healthy maternal gut microbiota is important, as it imprints progeny health and is a source of metabolites that shape progeny health outcomes.

## 4. Gestational Diabetes, Preeclampsia, Polycystic Ovary Syndrome and Metabolites

Some maternal metabolic phenotypes and diseases (that occur prior to, during and after pregnancy) are relevant in the context of maternal and child health and developmental programming. In gestation, several maternal diseases and compromised metabolic phenotypes may arise in women at greater risk for non-communicable disease, such as gestational diabetes, preeclampsia and polycystic ovary syndrome (PCOS).

### 4.1. Gestational Diabetes

The placenta alters its structure and physiology as it adapts, senses and responds to maternal milieu fluctuations, which may change the blood flow, nutrient supply to the fetus and secretion of signaling molecules [35]. In early gestation, the normalization of the mothers’ metabolic environment may reduce inflammation and oxidative stress that impair insulin sensitivity and beta cell physiology and predispose mothers and fetuses to become metabolically compromised [36,37,38,39,40,41]. During pregnancy, mothers adapt by becoming insulin resistant during early gestation, which increases up to twofold in late gestation [42]. The pre-conception body mass index (BMI) and gestational weight gain predict progeny growth [43]. Prior to conception, maternal obesity presents a risk for gestational diabetes [44], and it worsens inflammation, oxidative stress, hyperinsulinemia and insulin resistance [45], whereas the BMI shapes placental development, efficiency and oxygen delivery [44]. About 7% of women are affected by gestational diabetes, with a higher frequency in obese women characterized by systemic inflammation and insulin resistance [46]. Gestational diabetes intensifies the short-term maternal morbidity risk (e.g., preeclampsia and Cesarean or C-section delivery) and fetal morbidity risk (e.g., macrosomia, birth trauma and congenital defects (e.g., in the heart)).

In a recent study, 11 metabolites were identified to predict gestational diabetes, of which 6 were associated with glucose oxidation, fatty acid oxidation and amino acid catabolism, and 3 played a role in antioxidant pathways, potentially implicated in hyperglycemia-induced oxidative stress and inflammation [38,47,48,49,50]. This was aligned with the early pregnancy adaptions towards insulin resistance and diabetes in a longitudinal study that reported on 17 metabolites, with predictive values at ~16 weeks of gestation [50,51]. Apart from circulating maternal risk factors at ~12 weeks of pregnancy, plasma protein A, glycine, arginine and isovalerylcarnitine (pregnancy-associated) had 72% sensitivity, 80% specificity, 76% accuracy and a 0.83 receiver operating characteristic (ROC) area (a measure of diagnostic performance) [51]. ROC areas were reported from 0.641 to 0.858 for single tryptophan or purine urinary metabolites [52], with 71.7% predictive accuracy for dihydroorotate (a metabolite) [50]. Thus, metabolite profiles and patterns hold predictive potential for gestational diabetes, as they intersect oxidative stress, inflammation, hyperglycemia and insulin resistance.

### 4.2. Preeclampsia

Preeclampsia, an autoimmune gestational disease (in 3–5% of all pregnancies), is typified by varying degrees of placental malperfusion and antiangiogenic factor release into the bloodstream that result in maternal vascular endothelial injury, elevated blood pressure [11] and renal failure [53,54]. Preeclampsia is routinely diagnosed, and women are asymptomatic but have hypertension [55]. In early gestation, preeclampsia is linked to increased triglyceride concentrations and residual cholesterol [56]. Further, preeclampsia causes maternal and child morbidity and mortality, with ~42,000 of mothers dying from preeclampsia globally per annum [55]. Upon diagnosis, there is no cure until the mother gives birth. Lipid metabolite anomalies and altered lipid concentrations present risks for preeclampsia [11,57], as lipid and lipoprotein accumulation in the arterial wall may cause atheroma plaques to form, which may lead to atherosclerosis [58,59].

Inflammation is linked to embryonic implantation, pregnancy and delivery [11]. Lipids and lipid metabolism are key in the development of preeclampsia through the regulation of vascular and trophoblast physiology, and they trigger inflammation and ferroptosis [11]. Arachidonic acid (AA) and its metabolites are implicated in inflammation in preeclampsia [11]. Gestational inflammation equips mothers to bear their fetuses, but excessive inflammation may induce endothelial dysfunction and maternal vascular injury [60]. Through cyclooxygenase (COX) enzymes, AA is converted to prostacyclin and thromboxane, and in severe preeclampsia, there is an upsurge in platelet thromboxane synthesis, with maternal immune cells hypersecreting interlekin-6 (IL-6) and tumor necrosis factor alpha (TNF-α), which are proinflammatory cytokines [61]. Bioactive metabolite imbalances may induce ischemia and vascular stenosis, accounting for some preeclampsia symptoms (e.g., reduced uteroplacental blood flow, platelet aggregation and hypertension) [61,62]. The endogenous lipoxins, which are anti-inflammatory [63] lipid-based autacoids, are lipoxygenase-mediated biosynthesis products of AA [64]. Lipoxin A4 (LXA4) and its analogues are the stop signal for inflammation, and thus they may be key for inflammation in preeclampsia [64]. Metabolites that trigger gestational hyper-inflammation in preeclampsia need to be neutralized to prevent latent cardiovascular disease that may occur in mothers or be programmed in their children.

### 4.3. PCOS

PCOS presents similar to metabolic syndrome and is characterized by obesity, insulin resistance, dyslipidemia and hyperandrogenism [6], and it may confer these maternal risks to progeny. The microRNA miR-122 plays a role in the onset of insulin resistance through the inhibition of insulin-like growth factor 1 expression, which may relate to the role of insulin resistance in PCOS [65]. About 70% of PCOS patients display dyslipidemia with increased low-density lipoprotein cholesterol, very-low-density lipoprotein cholesterol, triglycerides and free fatty acid concentrations but reduced high-density lipoprotein cholesterol concentrations [66,67], reflecting susceptibility to non-communicable diseases.

PCOS is associated with miscarriages, gestational diabetes, preeclampsia, preterm birth, pregnancy-induced hypertension and C-section delivery [68,69,70,71]. Women with preeclampsia may deliver by C-section, which confers further risks for the mother and child [72]. Pregnancy complications, such as preeclampsia and gestational hypertension, confer risks for maternal and child morbidity [73]. Thus, gestational diabetes, preeclampsia and POCS are associated with non-communicable diseases and compromised metabolic phenotypes, and concomitant with other unfavorable sequelae, diminish maternal and child health during and subsequent to the gestational–fetal and lactational–neonatal life phases.

Several metabolites are implicated in the pathogenesis of PCOS. PCOS patients present with gut microbiota dysbiosis and aberrant metabolite profiles (e.g., short-chain fatty acids, bile acids, branched-chain amino acids (BCAAs), ceramides and trimethylamine N-oxide (TMAO)). In humans, bile acids are synthesized from cholesterol in the liver, can be re-metabolized by intestinal bacteria and ceramides, and are produced in various tissues [6]. BCAAs, such as essential amino acids (viz., leucine, isoleucine and valine) are derived from nutrition, as humans cannot synthesize them [74]. Because certain gut microbiota can synthesize some BCAAs in vivo, gut dysbiosis may be implicated in the pathogenesis of PCOS through the BCAA pathway [6]. TMAO is derived from trimethylamine (TMA), which is yielded from nutritional constituents in the intestine (e.g., L-carnitine and choline) [6]. After absorption in the intestine, TMA is transported via the portal vein to the liver, where flavin monooxygenase 3 (FMO3) produces TMAO [75] and is implicated in diabetes and cardiovascular disease [76,77].

## 5. Cancer

Metabolomic studies can unravel the complex association between metabolism and cancer risk to reveal metabolites and other biomarkers linked to cancer risk to gain insights into mechanisms that lead to the pathogenesis of cancer [78] to potentially prevent some cancers from progressing through early diagnosis and treatment. Metabolite families are potentially associated with risks for various types of cancer, and specific metabolites may reflect mechanisms underlying the carcinogenicity of cancer risk factors (e.g., obesity) [78].

There are limited cancer studies in the context of developmental programming, although the epigenome is integral for development and is associated with cancer. Further, the maternal-to-fetal transmission of cancer is rare due to placental barrier efficacy (syncytiotrophoblast cells) and immune surveillance [79]. However, the mother-to-infant transmission of uterine cervical cancer can occur in vaginal delivery; thus, in these mothers, C-section delivery is recommended [80]. In the transmission of uterine cervical tumors in mothers to the lungs of infants [80], the sequencing of paired tumors and normal tissue may be applied to diagnose cancer transmission (from mothers to infants) to determine the prevalence [80], and to identify metabolites that are biomarkers. In maternal–fetal transmission, cancer in infants was similar to that in mothers (i.e., a melanoma or leukemia/lymphoma), which suggests the ability of these cells to migrate, infiltrate and metastasize [81].

In pancreatic ductal adenocarcinoma (PDAC) serum and tissue, there were similar changes in differential metabolites (i.e., 17 metabolites were similarly altered) [82]. Further, in PDAC, the fatty acid (e.g., palmitic acid, myristic acid, linoleic acid, adrenic acid and arachidonic acid) and lipid levels increased, whereas the levels of several amino acids (e.g., proline, creatine, glutamate, isoleucine, taurine and tryptophan) and TCA products (e.g., succinate) decreased [82]. A biomarker panel with proline, creatine and palmitic acid was validated [82]. In PDAC and some cancer types, fatty acid and lipid upregulation may be due to an increase in lipases and de novo fatty acid synthesis [83,84]. A biomarker panel of proline, creatine and palmitic acid, with the addition of carbohydrate antigen 19-9 (CA19-9), was established to distinguish PDAC from benign pancreatic neoplasms [82], reflecting promise for PDAC diagnosis.

Of 50 metabolites studied, glutamine, butyrylcarnitine and lysophosphatidylcholine and three clusters of phosphatidylcholines (PCs) were negatively associated with most cancer types [78]. In addition, proline, decanoylcarnitine and a PC cluster were positively associated with most cancer types, whereas 10 metabolites were associated with specific cancer types—histidine was negatively associated with colorectal cancer risk, and a sphingomyelin cluster was positively associated with endometrial cancer risk but negatively associated with the risk of hepatocellular carcinoma [78]. Proline was positively associated with most cancer risks apart from breast cancer, and potentially hepatocellular carcinoma [78]. Altered glutamine and glutamate levels in people with cancer may signal enduring metabolism linked to the pathogenesis of cancer and could be early biomarkers for cancer risk (as changes in glutamine and glutamate may precede cancer development) [78]. Unraveling the metabolite profile shifts in various cancer types and severities may identify biomarkers and reveal therapeutic targets.

Gut microbiota also plays a role in cancer transmission from mother to child. Specific *Escherichia coli* strains that host the gene cluster *clb* (*clb* + *E. coli*) are involved in colibactin (a genotoxin) biosynthesis and associated with colorectal oncogenesis [85,86,87]. Colibactin-positive *E. coli* transmission may occur early in neonatal life and may be used in developing early preventive measures against colorectal cancer [88]. The incidence of being *clb*-positive was higher in infants delivered vaginally relative to those born by C-section [88]. Exclusively breastfed infants also had higher *clb* positivity compared to mixed-fed infants [88]. Further, colibactin-positive *E. coli* may be transmitted from mother to neonate very early in life or via close maternal contact [88]. Thus, reducing colibactin-positive *E. coli* transmission from mothers to infants may reduce colorectal cancer [88]. Further elucidation of the role of gut microbiota in shaping the risk for and onset of cancer, particularly in maternal transmission to children, presents an intriguing research avenue to pursue.

## 6. Mental Illness

Fetal programming enables psychopathology to be transmitted intergenerationally [89]. Maternal depression in gestation dysregulates maternal biological processes [89]. Neurochemical changes induce alterations in the fetal exposure to factors (e.g., cortisol) that can cross the placenta [89]. The programming of fetal molecular pathways may account for the mechanism of the progeny from depressed mothers developing varying reactivity to stress relative to those from healthy mothers [89], and concomitant with other genetic and environmental factors that shape development, may heighten the risk of the psychopathology of some progeny when they reach adolescence [89].

Antenatal depression symptoms are linked to a higher probability for preterm birth (<37 weeks of gestation) [90,91]. Perinatal mental disorders present a higher risk for child psychological and developmental impairment [92]. Antidepressants and smoking may be markers for more severe depression [93], and antenatal depression affected premature births and low birth weights when diagnosed as a disorder [91]; thus, the severity of maternal depression is a critical factor that influences preterm birth and low birth weights [92]. Maternal anorexia nervosa was also linked to low birth weight [94], whereas schizophrenia was linked to a higher risk for low birth weight, preterm birth, stillbirth and infant death within one year [95,96,97], which was due, in part, to poor nutrition, smoking, substance abuse and poverty [95]. Maternal mental illness thus influences birth outcomes and should be treated to minimize harmful effects transmitted to children. However, the medication used to treat mothers with mental illness may transmit undesirable metabolites, after drug metabolism, to fetuses and infants during gestation and lactation, respectively. More regulatory and toxicity tests should be conducted in a subset of patients in trials (i.e., pregnant and lactating mothers) to further discern the therapeutic and potential adverse effects of drugs in mothers and their progeny. This is increasingly important given the high global prevalence of mental illness, with even worse outcomes for mothers and children in low- and middle-income countries.

Maternal mental illness can have devastating consequences over the perinatal phase related to the care that the infant receives and the relations the infant forms [89] that are shaped early in life. During the perinatal phase, there is major brain development in progeny. In gestation (gestational–fetal life phase), the fetal genetic makeup interacts with maternal physiology, and after delivery (lactational–neonatal life phase), it is shaped by maternal nutrition, emotions and security [98,99]. Therefore, during the perinatal phase, maternal mental illness and disorders (e.g., depression and postpartum psychosis) may profoundly impact children’s cognitive, behavioral, emotional and social development [89]. Poor neonatal mental health outcomes deprive children of realizing their health, educational and economic potential, which feeds into the socio-economic determinants of mental illness that require treatment over the life course. Antenatal depression was linked to adolescent and young adult depression in progeny [100,101]. Depression in gestation is linked to low birth weight [91], with many possible mechanisms to account for how an adverse intrauterine milieu predisposes to diseases and disorders over the life course that can affect brain development, lead to inflammation, trigger epigenetic regulation and modulate the placental transport of stress hormones (e.g., cortisol [102]) to the fetus [89]. Thus, maternal mental health and well-being is critical for optimal child neurodevelopment, mental health and well-being.

Iron deficiencies could result in anemia, which may irreversibly impair mental and psychomotor development in children [103]. Untargeted metabolomic approaches utilizing broad metabolite panels are applied in mental health research to investigate associations and to inform new research avenues [104,105]. In a recent study, a metabolite panel was assessed in young adulthood to determine linkages between biomarkers and depression [106] as a substudy of a consortium that investigated the effects of the exposome (i.e., the internal (e.g., biomarkers) and external (e.g., physical environment and socioeconomic indicators) exposomes) on child mental health, from conception to adulthood (i.e., over the life course) [107]. The BCAA metabolites leucine and valine were negatively associated with depressive symptoms in young adults [106]. Further insights into the role of metabolites in maternal and child mental health and illness are important to better understand altered metabolite profiles that may present in mental illness.

The transcriptional co-activator peroxisome proliferator-activated receptor gamma coactivator 1 (PGC-1) regulates mitochondrial metabolism. The loss of PGC-1β in dendritic cells triggers increased inflammatory gene expression and reduced mitochondrial physiology gene expression (e.g., genes required for respiratory electron transport, the tricarboxylic (TCA) cycle and crista formation) [108]. Further, PGC-1β is required to maintain oxidative metabolism and the bioenergetic capacity, and to limit dendritic cell activation [108]. Loss of PGC-1β expression in dendritic cells resulted in an impairment in oxidative metabolism and higher glycolytic activity to compensate [108] for the energy deficiency in PGC-1β-deficient dendritic cells, resulting in AMPK activation, to lead to upregulated glycolysis and glucose uptake [108]. Although the glucose transporter (*Slc2a1*) mRNA expression was unchanged in PGC-1β-deficient dendritic cells under normoglycemia, glucose deprivation induced a higher fold change in *Slc2a1* mRNA expression, reflecting greater reliance on glucose [108]. Thus, in PGC-1β-deficient dendritic cells, oxidative metabolism is impaired due to the greater reliance on glycolysis [108].

## 7. Obesity

Obesity often precedes and is a risk factor for non-communicable diseases, and in mothers and children, obesity could predispose to diabetes, cardiovascular disease and some types of cancer. Obese pregnant women present with inflammation [109,110,111]. Zinc deficiency in children inhibits growth, which heightens the risk for morbidity and mortality [112]. High body and visceral fat in children and adults is linked to unfavorable plasma lipid profiles [113,114]. In obese children, BCAA concentrations were increased [115]. Infant growth patterns are associated with metabolite profiles [116,117]. At 3 months of age, the circulating phosphatidylcholine (PC) (20:4/18:0), PC plasmalogen (36:4) and sphingomyelin (SC) (d18:1/16:0) concentrations were linked to slow weight gain, whereas PC (18:1/16:0) and PC plasmalogen (34:1) were linked to rapid weight gain [116,117]. Further, LysoPC (22:2) and dimethylarginine predicted, albeit modestly, the truncal-to-peripheral skinfold ratio at 2 years of age [118]. At 3 months of age, PC 42:8 levels were higher in exclusively breastfed infants with high abdominal subcutaneous fat at 2 years of age, whereas, at 3 months, PC 38:3 levels were only higher in exclusively formula-fed infants with excessive abdominal subcutaneous fat at 2 years of age [119]. Several metabolites were linked to visceral fat at 2 years of age: lysophosopholipids (LysoPC 14:0; LysoPC 16:3; LysoPC 16:1; LysoPC 16:0; LysoPS 21:1; LysoPS 25:6 and LysoPA 23:1), dimethylarginine, diacylglycerol (DG 40:10) and sphingomyelin (SM 35:2; O2) [119]. Gestational weight gain (in mothers) and birth weights (children) are predictors of diabetes and cardiovascular disease in mothers and children, respectively. Metabolites that influence weight and growth trajectories may predict the onset of some non-communicable diseases.

LysoPA, LysoPE, LysoPG, LysoPS and dimethylarginine are metabolites linked to pro-inflammatory pathways in preadipocytes [120,121] and inflammation [122,123], primarily through interacting with peroxisome proliferator-activated receptor gamma (PPAR-γ) [124] and Toll-like receptor dimers [125] linked, via G-protein-coupled receptors, to the pathogenesis of cardiovascular disease [126]. The metabolites identified at 3 months of age have roles in the development of adiposity and inflammation, which may be initiated early in life [119]. The link of the metabolite profile at 3 months to body composition at 2 years was dependent on the infant feeding type [119]. The feeding type and composition of milk impact infants’ metabolite profiles [116,117,118,127], and metabolite profile differences in early life are likely implicated in the adiposity differences in breastfed and formula-fed infants [119]. This distinctive metabolite profile, shaped by nutrition over the early life course, reveals associations with adiposity and inflammation that may trigger the onset of non-communicable diseases later in life.

A metabolically unhealthy obese phenotype in Chinese adolescents was linked to 9 metabolites that included saturated fatty acids: palmitic acid and stearic acid; amino acids: asparagine, alanine and isoleucine; and other metabolites: glycolic acid, phosphate 3-hydroxypropionic acid and 2-hydroxypentanoic acid [7], whereas a metabolically unhealthy obese state was linked to 19 metabolites, which overlapped with the same metabolites, apart from phosphate 3-hydroxypropionic, but also included 2-hydroxybutanoic acid, 3-hydroxypropionic acid, 5-methyluridine, acetophenone, beta-gentiobiose, cyanoalanine, furoylglycine, galactinol, glycerol-alpha-phosphate, isocitric acid minor, salicylaldehyde and shikimic acid [7]. In metabolically unhealthy obese phenotypes, the major metabolic pathways were involved in fatty acid biosynthesis, fatty acid elongation in mitochondria, propanoate metabolism, glyoxylate and dicarboxylate metabolism, fatty acid metabolism and phenylalanine, tyrosine and tryptophan biosynthesis (only in boys) [7]. Interestingly, the fasting BCAA and 2-hydroxybutyric concentrations may predict insulin resistance in adolescents, which could prevent the onset of non-communicable diseases in adulthood [7], with 2-hydroxybutyric acid linked to beta cell dysfunction, which serves as a biomarker for the early detection of insulin resistance and diabetes [128]. The metabolites linked to the metabolically healthy obese phenotype may reflect the early risk metabolite profile prior to the onset of cardiovascular disease [7]. Thus, in obese adolescents, specific metabolites may predict pre-disease states or risks for diabetes and cardiovascular disease. Further studies on the altered metabolite profiles in overweight/obese children, adolescents and adults from various ethnicities will provide insights for combating obesity for the prevention of non-communicable diseases.

## 8. Maternal and Child Health, Non-Communicable Diseases and Metabolites

Mothers shape the health outcomes of their children during the gestational–fetal and lactational–neonatal phases that chart their children’s health and disease trajectories. An early life milieu that is unfavorable has lasting health and disease consequences that increase susceptibility to non-communicable diseases through epigenetic [129,130] and developmental programming mechanisms [1]. Healthy mothers shape favorable child outcomes, which are shaped by maternal nutrition and healthy maternal phenotypes over these critical phases.

Maternal gestational diseases (and metabolic phenotypes), such as gestational diabetes (a risk for diabetes), preeclampsia (a risk for cardiovascular disease) and PCOS (a risk for metabolic syndrome), increase the susceptibility of the mother (due to a compromised metabolic state (i.e., gestational disease or disorder)) and child (through developmental programming) to develop non-communicable diseases. Further, maternal cancer, mental illness and obesity provide an unfavorable milieu that may induce fetal programming, thereby perpetuating these diseases and unfavorable metabolic sequalae to progeny, which compromises children’s growth, development, physiology and metabolism (i.e., health) over their life course. Metabolites, in the gestational–fetal and lactational–neonatal phases, are typically derived from the metabolism of food, which sustains the progeny through the gestational diet or breastmilk (and/or formula), respectively, or aberrant signaling pathways and physiology. Metabolites thus serve as predictors of diseases and may present in pre-disease phases (e.g., prediabetes) or in risk factors and compromised metabolic states (e.g., obesity) to identify the progression of metabolic aberrances that lead to disease manifestation. Disease-predictive metabolites can thus be utilized for early detection in the pathogenesis of non-communicable (and other) diseases, and to identify patterns for disease prevention and treatment.

Gestational diseases compromise maternal and child health and may lead to the onset of maternal and child non-communicable diseases (i.e., with origins in the critical gestational–fetal phase). This can be extended into the lactational–neonatal phase, when both mother and child remain vulnerable to the onset of non-communicable diseases. In progeny, the fetal and neonatal life stages are critical periods for developmental programming.

### 8.1. Gestational Origins and Interplay of Non-Communicable Diseases

**Figure 1:**
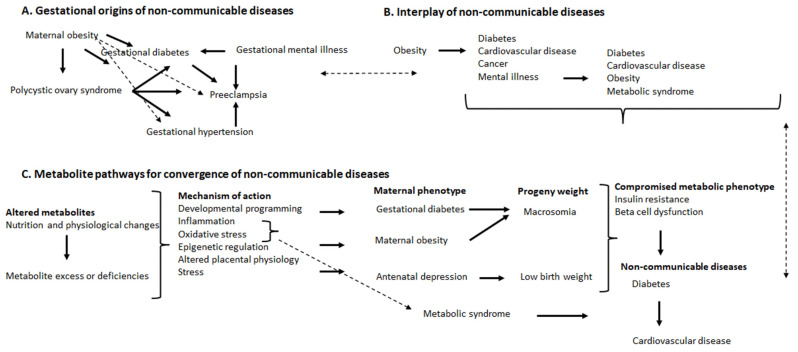
Gestational origins and interplay of non-communicable diseases and metabolite pathways for convergence of non-communicable diseases.

PCOS increases the risk for gestational hypertension, preeclampsia and gestational diabetes [69,70,71], which increases the risk for developing diabetes [131,132] and cardiovascular disease [133,134,135] in mothers and their progeny [68], reflecting a developmental programming link. PCOS and gestational diabetes increase the risk for neonatal hypoglycemia [136]. There is a higher risk in overweight/obese women (maternal obesity) of developing PCOS, gestational diabetes, gestational hypertension and preeclampsia, which present greater risks for mothers and their progeny of developing diabetes and cardiovascular disease [44,137,138] (Figure 1A). An elevated pre-gestational BMI may predispose to gestational hypertension, which is implicated in the pathogenesis of preeclampsia and increases the risk for placental abruption [139]. Gestational diabetes, gestational hypertension and gestational mental illness are risk factors for preeclampsia (Figure 1A). These diseases affect maternal and child health and often lead to non-communicable diseases later in life. Further, women with gestational mental illness are at higher risk for gestational diabetes and preeclampsia [140] (Figure 1A). These gestational diseases transition into non-communicable diseases in adulthood (increases risk), and, similarly, adults with non-communicable diseases can increase the risk and intensify the severity of gestational non-communicable diseases and disorders with adverse consequences for the mother and child (Figure 1A,B).

For the interplay of non-communicable diseases, in adulthood, obesity is a risk for and is closely associated with diabetes, cardiovascular disease, some types of cancer and mental illness [141,142,143,144,145] (Figure 1B). Mental illness, in turn, is associated with diabetes, cardiovascular disease, obesity and metabolic syndrome [146,147,148,149,150,151] (Figure 1B). Further, gestational diseases and disorders confer greater risk for the mother and child to develop non-communicable diseases, which links the gestational origins of disease, developmental programming, maternal and child health and non-communicable diseases.

### 8.2. Metabolite Pathways and Convergence of Non-Communicable Diseases

Metabolites play a role in shaping maternal and child health, and altered metabolite profiles (Figure 1C and Table 1) present in diseases and may lead to the onset of non-communicable diseases; therefore, more studies are required to provide insights on the interplay of maternal and child health and non-communicable diseases. Maternal nutrition and lifestyle influence fetal growth, development, health and well-being [152], and infant nutrition can, in turn, alter metabolite profiles [153]. During gestation and lactation, metabolite profiles may also be altered by the intake of drugs, physical activity, the consumption of iodized salt, supplements, and nervine beverages and smoking, which are all correlated with specific metabolites in neonates [153]. Metabolites are altered by nutrition and physiological aberrations that result in excesses or deficiencies (Figure 1C). In mothers, elevated glucose (hyperglycemia), excess saturated fatty acids (e.g., palmitic acid and stearic acid) and amino acid deficiencies (e.g., reduced alanine and serine), during gestation and lactation, elicit undesirable sequelae to progeny (developmental programming) while also potentially compromising maternal health during these critical windows, should metabolic adaptations be inadequate. Apart from developmental programming, some of the mechanisms implicated in the pathogenesis of gestational and non-communicable diseases are inflammation and oxidative stress, epigenetic regulation, altered placental physiology and stress [36,49,109,110,154,155] (Figure 1C). Inflammation and oxidative stress, which is systemic in non-communicable diseases, can also be localized in the placenta (maternal obesity) and brain (mental illness) and present variable birth outcomes and weights (e.g., macrosomia due to gestational diabetes or maternal obesity, or low birth weights due to protein deficiency or placental insufficiency) [156,157,158] (Figure 1C). Gestational mental illnesses are associated with low birth weights [91,92,94], which compromise brain development, trigger inflammation, induce epigenetic regulation and alter the placental transport of cortisol (a stress hormone) to the fetus [89,102]. Altered maternal and placental physiology in maternal obesity contribute to fetal programming and adverse neurodevelopmental outcomes in progeny. The placenta mediates the adverse effects of maternal obesity on fetal brain development, which include inflammation, fetal immune activation, lipotoxicity, elevated oxidative stress and reduced antioxidant capacity [159]. Low birth weights and macrosomia are implicated in the development of diabetes and cardiovascular disease later in life [160,161,162,163,164]. Macrosomia due to in utero exposure to excess glucose [19] or saturated fatty acids and maternal obesity also lead to diabetes and cardiovascular disease [161,165,166,167]. Metabolic syndrome, which is characterized by inflammation and oxidative stress, presents a risk for developing diabetes and cardiovascular disease [168,169,170] (Figure 1C).

Macronutrients are digested and metabolized into monomers and other metabolites that elicit effects, systemically, and in organs, to shape health and disease. Systemic inflammation presents in diabetes, cardiovascular disease, obesity and metabolic syndrome. Diabetes heightens the risk for cardiovascular disease, which is intensified by hypertension and inflammation, oxidative stress and fibrosis to induce microvascular and macrovascular diabetic complications resulting in vascular remodelling and dysfunction in response to hypertension [171]. Altered metabolite profiles induce inflammation, oxidative stress, elevated stress (e.g., increased cortisol concentrations), altered placental transport and physiology, epigenetic regulation and developmental programming (i.e., exposure to excess glucose in fetal and/or early neonatal life), leading to a compromised metabolic phenotype (e.g., insulin resistance and beta cell dysfunction) that progresses to diabetes, and with severe diabetes, micro- and macrovascular events that lead to cardiovascular disease (Figure 1C). Progeny of overweight/obese mothers are prone to develop cardiovascular irregularities [172], with obese pregnancies linked to greater risk for cardiovascular disease in progeny in adulthood [173]. These bidirectional pathways are interrelated, as the altered metabolites that lead to diabetes and cardiovascular disease (Figure 1C) are linked to the interplay of non-communicable diseases (Figure 1B), which, in turn, can be linked to the gestational origins of non-communicable diseases (Figure 1A).

Table 1 lists some metabolites implicated in maternal and child health and non-communicable diseases. These metabolites vary across diseases and during the progression of diseases (e.g., from prediabetes to diabetes). Further, some diseases with gestational (maternal) origin may be conferred to children and become more overt in mothers postpartum. For example, gestational diabetes (in mothers) may confer a diabetic phenotype to children through the transmission of one or more undesirable sequelae (e.g., hyperglycemia, beta cell dysfunction, glucose intolerance and/or insulin resistance), and the mothers may develop diabetes postpartum.

**Table 1:** Potential metabolite biomarkers implicated in maternal and child health non-communicable diseases.

Disease	Increase (Supplementation)	Decrease (Deficiency)
Gestational diabetes	Arginine [51]	3-hydroxy-isovalerylcarnitine, glycine [51].
Diabetes	7-methylguanine, aspartate, cystine, glutamate, glutamic acid; glutamine, glycine, isoleucine, leucine, lysine, N-acetylcysteine, N-acetyl-D-galactosamine, phenylalanine, putrescine, trimethylamine N-oxide, tryptophan, tyrosine, valine [4,22,23,24,28,76,174].	Alanine, serine [23].
Preeclampsia	3-phosphoglycerate, arachidonic acid, glutamate; palmitoleic acid, sphingomyelins (SMs): SM C28:1, SM C30:1; oxidized phospholipids (PLs): OxPC, OxPI, OxPE; xanthine, C30:1, C32:1, C33:2, C34:2, C38:1, fatty acid esters of hydroxy fatty acid (FAHFA): (C18:0), PCs, PE, LysoPC, LysoPEs [11,175].	Lipoxin A4 [64].
Cardiovascular disease	Betaine, choline, trimethylamine [77].	Creatine, glutamate, isoleucine, proline, succinate, taurine, tryptophane [82].
PCOS	Isoleucine, leucine, trimethylamine N-oxide (TMAO) valine [6,176,177].	
Mental illness	Isoleucine [104].	Leucine, valine [106,178,179],
Cancer	PDAC Adrenic acid, arachidonic acid, linoleic acid, myristic acid, palmitic acid [82]. Colorectal Proline [78].	creatine, glutamate, isoleucine, proline, succinate, taurine, tryptophane [82]. Histidine [78]
Obesity	PC (18:1/16:0), PC plasmalogen (34:1) [116,117]. Elevated in children with high visceral fat Lysophosopholipids (LysoPC 14:0, LysoPC 16:3, LysoPC 16:1, LysoPC 16:0, LysoPS 21:1, LysoPS 25:6 and LysoPA 23:1), dimethylarginine, diacylglycerol (DG 40:10), sphingomyelin (SM 35:2; O2) [119]. Metabolically unhealthy obese phenotype Palmitic acid; stearic acid; asparagine; alanine; isoleucine; glycolic acid; * phosphate 3-hydroxypropionic acid; 2-hydroxypentanoic acid [7]. Metabolically unhealthy obese status 2-hydroxybutanoic acid; 3-hydroxypropionic acid; 5-methyluridine; acetophenone; beta-gentiobiose; cyanoalanine; furoylglycine; galactinol; glycerol-alpha-phosphate; isocitric acid minor; salicylaldehyde; shikimic acid [7].	Elevated in children with low visceral fat Lysophosphatidylserine (LysoPSs), lysophosphosphatidic acid (LysoPA), sphingomyelin (SM) [119].

* Not in metabolically unhealthy obese states.

Metabolites can serve as biomarkers to predict pre-disease states and the risk factors for and onset of non-communicable diseases, and with earlier detection, allow for better prognosis. Further, metabolite profiles have distinctive signatures in health and disease and can therefore be applied to monitor health and disease progression in mothers and children. Thus, metabolites in gestation can help to predict diseases in gestation (and fetal life) and that extend into lactation (and neonatal life) and over the life course, and they can be an early signal to focus and implement strategies for the prevention of the onset of non-communicable diseases in mothers and children. Many metabolite profiles are altered in gestational diseases (which imprint mothers and their children), pre-diseased states and non-communicable diseases (Table 1) and further investigation will provide insights into the different metabolic states and phenotypes that present. Metabolite profiles and patterns hold predictive potential for gestational and non-communicable diseases that intersect to better define their pathogenesis and potentially yield clues for therapeutic targets.

## 9. Conclusions

The gestational–fetal and lactational–neonatal phases are critical periods that impact maternal health and well-being and shape progeny (child) health outcomes. Exposure to metabolites over these life phases, when mothers and their children are most vulnerable, are integral for maintaining physiology, and imbalances lead to undesirable effects that can manifest as non-communicable diseases, such as diabetes, cardiovascular disease, cancer, mental illness and obesity. Given metabolites’ role and interplay in and influence on physiological systems and signaling pathways, investigating the dynamic and evolving roles of metabolites in health and disease remains intriguing for the discovery of biomarkers and novel therapeutic agents, particularly in the context of maintaining, improving and optimizing maternal and child health.
